# Performance of Two AI Approaches in ASReview Compared With Manual Screening for Dementia Care Literature Screening: Comparative Analysis

**DOI:** 10.2196/84989

**Published:** 2026-08-20

**Authors:** Dirk Steijger, Stella Thissen, Sil Aarts, Hilde Verbeek, Marjolein E de Vugt, Hannah Christie

**Affiliations:** 1 Department of Psychiatry and Neuropsychology, Mental Health and Neuroscience Research Institute, Faculty of Health, Medicine and Life Sciences, Maastricht University, Dr Tanslaan, 12, Maastricht, 6229ET, The Netherlands, 31 612365497; 2 Department of Health Service Research, CAPHRI Care and Public Health Research Institute, Faculty of Health Medicine and Life Sciences, Maastricht University, Maastricht, The Netherlands; 3 The Living Lab in Ageing & Long-Term Care, Maastricht, The Netherlands; 4 Department of Primary and Community care, Research Institute for Medical Innovation, Radboud university medical center, Nijmegen, The Netherlands; 5 School of Population Health, Royal College of Surgeons, Dublin, Ireland

**Keywords:** ASReview, dementia, literature screening, AI, information retrieval

## Abstract

**Background:**

Literature reviews rely on rigorous title and abstract screening by researchers, which is time-consuming. AI-assisted literature screening tools have been proposed to improve efficiency by prioritizing titles and abstracts with the highest likelihood of meeting the inclusion criteria, thereby reducing the need to screen all records.

**Objective:**

This study aims to evaluate the performance of two AI-assisted screening approaches in ASReview (version 1.3; Department of Methodology and Statistics, Utrecht University) compared with manual title and abstract screening in a previously completed and published scoping review on how AI can support the quality of life in people with dementia.

**Methods:**

This study used a dataset of 4690 titles and abstracts from a published scoping review. The manual screening decisions of the scoping review served as the reference standard. Both ASReview approaches were applied by the same author who conducted the majority of the original manual title and abstract screening. Approach A used a simpler model with minimal prior input, whereas approach B used a more advanced model with a larger training set. Both ASReview approaches applied predefined stopping rules: (1) more than 10% of the dataset to be screened; and (2) 50 consecutive irrelevant titles and abstracts. Performance was evaluated in terms of sensitivity, specificity, precision, accuracy, and screening time. 95% CIs were calculated for sensitivity, specificity, precision, and accuracy. Agreement between manual and ASReview approaches was assessed using the Cohen κ, and differences in how manual and both ASReview approaches classified titles and abstracts were examined using the McNemar test. Performance and agreement were calculated at two levels: (1) after title and abstract screening and (2) after full-text inclusion.

**Results:**

Manual screening identified 283 titles and abstracts for full-text review and resulted in 30 final included studies, requiring 19 hours. Of the 4690 titles and abstracts, approach A screened 830 (17.7%) in 4.3 hours and retrieved 16 of the 30 (sensitivity 0.53, 95% CI 0.36‐0.70) final included studies, whereas approach B screened 798 (17.0%) in 5.5 hours and retrieved 21 of the 30 (sensitivity 0.70, 95% CI 0.52‐0.83) final included studies. Although both ASReview approaches showed high specificity and accuracy, these metrics should be interpreted cautiously because the dataset was highly imbalanced and contained relatively few relevant titles and abstracts. McNemar tests showed significant directional imbalance (*P*<.001): ASReview missed more manually selected titles and abstracts at level 1, whereas at level 2, ASReview more often labeled titles and abstracts not included in the final review as relevant.

**Conclusions:**

ASReview can support workload reduction in title and abstract screening, but the evaluated ASReview approaches did not retrieve all final included studies from the original dementia care scoping review. These findings suggest that the evaluated ASReview configurations may be insufficient for reviews in which near-complete retrieval of relevant evidence is required.

## Introduction

In health research, evidence synthesis is used to integrate and summarize existing literature and to inform future research, guidelines, policy, and decision-making [1]. Title and abstract screening is a tedious but vital step in this process [2]. The purpose of title and abstract screening is to identify those that are potentially eligible for full-text assessment and to exclude those that clearly do not meet the eligibility criteria [2]. Although title and abstract screening is only 1 component of the evidence synthesis process, it is a key step in determining which records proceed to full-text review. Overlooking relevant titles and abstracts during screening can introduce bias and affect the completeness of the evidence base [1]. Because the title and abstract screening phase often involves a large number of titles and abstracts, it is time-consuming and cognitively demanding [3]. Therefore, efficient and innovative approaches to support the title and abstract screening process are needed.

To streamline the title and abstract screening process, various AI-assisted screening tools have emerged [4-8]. These AI-assisted screening tools may use techniques such as text mining, machine learning, natural language processing, and deep learning to identify and actively learn patterns in titles and abstracts and prioritize titles and abstracts that are more likely to be relevant. AI-assisted screening can be implemented in specialized screening tools, such as ASReview (version 1.3; Department of Methodology and Statistics, Utrecht University) [6], and Abstrackr [9] or as AI-supported functions within broader review management platforms, such as DistillerSR [10], Covidence [11], and Rayyan [12]. These AI-assisted screening tools are intended to support reviewer decision-making by prioritizing titles and abstracts based on predicted relevance [5]. By identifying likely relevant studies early, these AI-assisted screening tools could potentially allow reviewers to screen only a subset of the dataset, thereby reducing workload.

Previous studies evaluating AI-assisted screening tools in health research have shown that these tools can potentially reduce screening time while maintaining the ability to identify relevant titles and abstracts [9,13-17]. Focusing specifically on ASReview, a widely used open-source AI-assisted screening tool [18], previous evaluations have reported promising performance in different review contexts [14,19-23]. The performance of AI-assisted screening tools depends on the effectiveness of the underlying AI techniques and algorithms, the quality and quantity of data used for training, and the degree of human involvement [7,24,25]. Therefore, further evaluations are needed to understand how ASReview performs in title and abstract screening when different model configurations are applied across different review contexts.

Given this dependence on context, the intersection of dementia care, quality of life, and AI provides a relevant context for further evaluation. This intersection provides a useful starting point for examining how ASReview performs in a review area that combines dementia care research with technology-oriented literature. To our knowledge, ASReview has not yet been evaluated in such a dementia care literature review context. Therefore, this study aimed to evaluate the performance of two AI-assisted screening approaches within ASReview compared with manual title and abstract screening in a previously completed and published scoping review on how AI can support quality of life in people with dementia [26]. Specifically, this study examined the extent to which both ASReview approaches reduced screening workload and identified the same titles and abstracts as included via manual screening in the scoping review. By comparing title and abstract screening in ASReview with a manual screening process, this study adds empirical evidence on the practical use of AI-assisted screening in a real-world dementia care review context.

## Methods

### Study Design

This study was designed as a comparative methodological evaluation of two AI-assisted screening approaches within ASReview compared with the manual screening approach used in a previously published scoping review on how AI can support quality of life in people with dementia [26]. Reporting of the present study was guided by the PRISMA-trAIce (Preferred Reporting Items for Systematic Reviews and Meta-Analyses–Transparent Reporting of AI-Assisted Evidence Synthesis) guidelines for the transparent reporting of AI-assisted evidence synthesis, where applicable [27].

### Unit of Analysis, Data Source, and Study Size

The unit of analysis was the individual title and abstract record. The data were derived from the title and abstract dataset of the original scoping review, including both the manual screening decisions and the final full-text inclusion decisions. Manual screening and inclusion decisions from the original review served as the reference standard against which the two ASReview approaches were evaluated. The study size corresponded to that of the full screening dataset from the original scoping review. After deduplication and preparation for screening, the dataset consisted of 4690 titles and abstracts, of which 283 were selected for full-text review and 30 were included in the final review.

### Compared Approaches

#### Manual Approach

The manual screening approach was performed as part of a previously published scoping review [26]. In brief, the original review searched PubMed, Scopus, the Association for Computing Machinery Digital Library, and Google Scholar for studies published between 2010 and January 2024 on AI-based approaches that could support the quality of life of people with dementia. Eligible studies involved people with at least suspected dementia, evaluated an AI-based approach to the daily living of people with dementia, and reported original empirical research. Studies were excluded if they focused on diagnostic or acute care applications, if they were not available in English or Dutch, or if the full text was unavailable.

After deduplication, records were imported into Rayyan and screened, without the use of the AI function in Rayyan, against the eligibility criteria. Initially, 2 reviewers (DS with 5 years of research experience and the other reviewer HC with 10 years of research experience) independently screened 10% of the titles and abstracts from the scientific databases. As the interrater agreement exceeded the predefined threshold (80%), 1 reviewer (DS) continued screening the remaining titles and abstracts. The same procedure was applied to full-text screening. For the Google Scholar gray literature search, 1 reviewer (DS) assessed the first 300 results. However, no Google Scholar records were included in the final review or in the dataset used for the present ASReview evaluation. A detailed description of the search strategy, eligibility criteria, and screening process is reported in the original scoping review [26]. The resulting manual title and abstract screening decisions and final full-text inclusion decisions were used as the reference standards for the present methodological evaluation.

#### ASReview

ASReview is an open-source AI-assisted screening tool that uses active learning to prioritize titles and abstracts according to their predicted relevance [6]. Before the title and abstract screening starts, ASReview requires prior knowledge consisting of titles and abstracts labeled as relevant and irrelevant by the reviewer. Based on this prior knowledge, the tool ranks the remaining titles and abstracts from highest to lowest predicted relevance. The reviewer then screens the highest-ranked title and abstract and labels it as relevant or irrelevant according to the eligibility criteria. After each reviewer’s decision, ASReview updates the ranking and presents the next title and abstract with the highest predicted relevance. This researcher-in-the-loop process continues until a predefined stopping rule is reached. ASReview allows reviewers to preselect different model configurations, including feature extraction methods (ie, Doc2Vec, embedding inverse document frequency, term frequency–inverse document frequency [TF-IDF], and Sentence-Bidirectional Encoder Representations from Transformers [sBERT]) and classifiers (Naïve Bayes, support vector machines, deep neural network, logistic regression, long short-term memory, and random forest) [6]. In the present study, ASReview was applied to the same dataset and eligibility criteria as the manual screening approach. This allowed a direct comparison between the original manual screening decisions and the screening decisions generated through two AI-assisted ASReview configurations. The two ASReview configurations were based on recommendations from ASReview’s model selection guide and the SAFE (Screen, Apply, Find, Evaluate) procedure [24,25]: a default configuration and a custom configuration.

#### Approach A: Default Configuration

Approach A was included as a baseline AI-assisted screening approach because it reflects ASReview’s default configuration and requires minimal prior-knowledge input from the reviewer [28,29]. It used term TF-IDF for feature extraction, combined with a Naïve Bayes classifier. The query strategy was set to maximum, and the balance strategy was set to dynamic resampling (double). TF-IDF represents titles and abstracts based on word frequencies, while Naïve Bayes uses these patterns to estimate the likelihood of relevance. Four titles and abstracts, consisting of 1 relevant and 3 irrelevant examples, were used as prior knowledge. The prior-knowledge titles and abstracts were included in the analysis as classified titles and abstracts by ASReview.

#### Approach B: Custom Configuration

Approach B was included to evaluate whether a more semantically rich model configuration, combined with a larger prior knowledge set, would improve the retrieval of relevant titles and abstracts compared with the default configuration. Approach B used sBERT for feature extraction, combined with a fully connected neural network classifier. In ASReview version 1.3, sBERT-based feature extraction required an extension package. The exact extension package name and version used in the present analysis were not retained. The query strategy was set to maximum, and the balance strategy used dynamic resampling (double). sBERT represents titles and abstracts as dense semantic vectors that capture contextual meaning beyond individual word frequencies, while the neural network classifier uses these representations to estimate the likelihood of relevance. Prior knowledge consisted of 47 titles and abstracts, corresponding to approximately 1% of the original dataset. This set included 2 relevant titles and abstracts randomly selected from the 30 final included titles and abstracts and 45 irrelevant titles and abstracts randomly selected from the titles and abstracts that were excluded for full-text screening in the original review. This ensured that the prior knowledge set contained both relevant and irrelevant titles and abstracts, consistent with the SAFE procedure’s recommendation to start with a labeled training set of approximately 1% of the dataset containing at least 1 relevant and 1 irrelevant title and abstract [25]. Prior knowledge titles and abstracts were included in the analysis as classified titles and abstracts by ASReview.

### Screening Procedure

The screening procedure and stopping rule were informed by selected elements of the SAFE procedure [6,24,30]. The SAFE procedure provides practical guidance for active learning–based screening, including the use of prior knowledge and stopping heuristics that combine a minimum screening proportion with a run of consecutive irrelevant titles and abstracts. Because the present study was a retrospective methodological evaluation rather than a live review selection process, the full SAFE procedure was not implemented. Instead, selected SAFE elements were used to define the prior-knowledge strategy and stopping rule for the ASReview screening approaches.

For the present study, 2 separate ASReview projects were created in the ASReview environment, 1 for approach A and 1 for approach B. For each project, the complete title and abstract dataset from the original scoping review were exported from Rayyan and uploaded to ASReview. Approach A and approach B were conducted as independent comparative screening projects. The screening decisions from approach A were not used as training data for approach B.

To ensure consistency in the application of the eligibility criteria, the same reviewer who conducted the majority of the title and abstract screening in the original scoping review (DS) also screened the titles and abstracts presented by ASReview for each approach. The same eligibility criteria as in the original scoping review were applied. Titles and abstracts were labeled as relevant or irrelevant independently for approach A and approach B.

Screening continued until the predefined stopping rule was met. This stopping rule required that 2 conditions were fulfilled: first, at least 10% of the complete dataset had to be screened; second, after this minimum threshold had been reached, screening continued until 50 consecutive titles and abstracts had been labeled as irrelevant by the reviewer. The threshold of 50 consecutive irrelevant titles and abstracts was selected as a pragmatic stopping criterion. The authors acknowledge that more conservative thresholds, such as 100 consecutive irrelevant titles and abstracts, may increase sensitivity by extending the screening process. The same stopping rule was applied to both ASReview approaches. Because each ASReview configuration generated a different ranking of titles and abstracts, the stopping rule was reached after a different number of screened titles and abstracts in each approach. After stopping, the titles and abstracts labeled as relevant by each ASReview approach were compared with the manual title and abstract screening decisions and final inclusion decisions from the original scoping review.

### Performance Outcomes

To assess performance, both ASReview approaches were compared with manual screening using sensitivity, specificity, precision, accuracy, screening time, and agreement. Sensitivity was used to assess the proportion of manually selected titles and abstracts that were also identified by each ASReview approach. Specificity was used to assess the proportion of manually excluded titles and abstracts that were also excluded by ASReview. Precision was used to assess the proportion of titles and abstracts labeled as relevant by each ASReview approach that were also relevant according to the manual approach. Accuracy was used to assess the overall proportion of titles and abstracts that were correctly classified by each ASReview approach, including both records considered relevant and records considered irrelevant according to the manual approach. Agreement between each ASReview approach and the manual approach was measured using the Cohen κ and observed agreement (*P*_o_) [31], and the McNemar test was used to assess directional imbalance in discordant classifications [32]. Total screening time was estimated using the time-tracking information displayed within Rayyan for the manual approach and within ASReview for both AI-assisted approaches. Screening time did not include full-text screening or data extraction. False negatives were examined descriptively to explore potential screening-related selection bias by assessing whether missed titles and abstracts shared common characteristics, such as dementia care context, AI methods, or failure to report AI performance. The evaluation level at which each metric was calculated is described in the Data Analysis section.

### Statistical Analysis

Performance was assessed at 2 levels. At level 1, manual title and abstract screening decisions were used as the reference standard. Thus, titles and abstracts selected for full-text review during manual screening were considered relevant, whereas titles and abstracts excluded during manual title and abstract screening were considered irrelevant. At this level, sensitivity, Cohen κ, observed agreement, and the McNemar test were calculated.

At level 2, the final full-text inclusion decisions from the original scoping review were used as the reference standard: titles and abstracts included in the final review were considered relevant, whereas all other records were considered irrelevant. At this level, sensitivity, specificity, precision, accuracy, Cohen κ, observed agreement, and the McNemar test were calculated. This approach aligns with prior research, suggesting that although ASReview retrieves fewer abstracts, a higher proportion may be included in the final inclusion set [25,33]. For binary classification metrics, 95% CIs were calculated using Wilson score intervals [34]. At both levels, prior knowledge titles and abstracts were included in the statistical analysis of the performance metrics.

Screening time referred only to the title and abstract screening process and did not include full-text screening (during the manual approach). For all approaches, total screening time was recorded in minutes at the end of the screening process.

## Results

**Figure 1.**
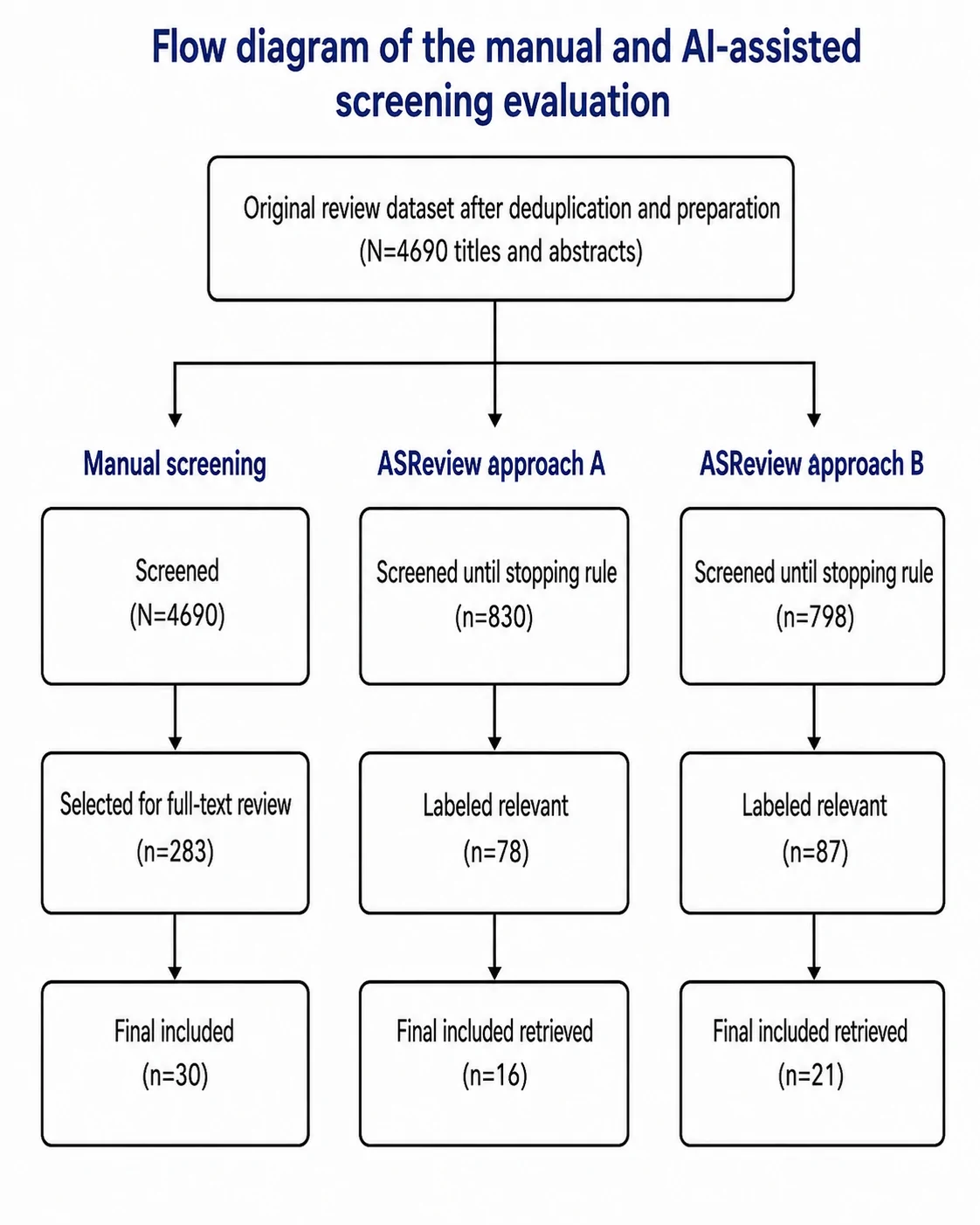
Flow diagram of titles and abstracts through two AI-assisted literature screening approaches within ASReview compared with manual title and abstract screening from a dementia care scoping review. The figure summarizes how the dataset from the original dementia care scoping review was screened manually and with two AI-assisted approaches within ASReview. After deduplication and preparation, the dataset consisted of 4690 titles and abstracts. Manual screening assessed all 4690 titles and abstracts, selected 283 for full-text review, and resulted in 30 final included studies. Approach A screened 830 titles and abstracts until the predefined stopping rule was reached, labeled 78 titles and abstracts as relevant, and retrieved 16 of the 30 final included studies. Approach B screened 798 titles and abstracts until the predefined stopping rule was reached, labeled 87 titles and abstracts as relevant, and retrieved 21 of the 30 final included studies.

Figure 1 summarizes the flow of titles and abstracts through the manual screening approach and the two ASReview approaches. The manual screening approach screened a highly imbalanced dataset of 4690 abstracts, identified 283 abstracts for full-text review, and included 30 final studies, requiring 19 hours. Approach A achieved 50 consecutive irrelevant abstracts and thus stopped screening at 830 (17.7%) abstracts in 4.3 hours, with a sensitivity of 0.16 at level 1 and 0.53 at level 2 (16 abstracts), specificity of 0.99, precision of 0.21, and accuracy of 0.98 at level 2. Approach B achieved 50 consecutive irrelevant abstracts and thus stopped screening at 798 (17.0%) abstracts in 5.5 hours, achieving higher sensitivity at both level 1: 0.23 and level 2: 0.70 (21 abstracts), specificity of 0.99, precision of 0.24, and accuracy of 0.98 at level 2. Both ASReview approaches reduced screening time compared with manual screening. At the final full-text inclusion decisions from the original scoping review stage, sensitivity was higher for approach B than for approach A (0.70 vs 0.53; an absolute difference of 0.17). The performance metrics are shown in Table 1. The 95% CIs indicate uncertainty around the sensitivity and precision estimates, particularly at level 2. For approach B, level 2 sensitivity was 0.70 (95% CI 0.52‐0.83) and level 2 precision was 0.24 (95% CI 0.16‐0.34).

**Table 1.** Performance of two AI-assisted literature screening approaches within ASReview compared with manual title and abstract screening from a dementia care scoping review.^a^

Approach	Sensitivity (level 1, 95% CI)^b^^,^^c^	Sensitivity (level 2, 95% CI)^b^^,^^d^	Specificity (level 2, 95% CI)^b^^,^^d^	Precision (level 2, 95% CI)^b^^,^^d^	Accuracy (level 2, 95% CI)^b^^,^^d^	Screened titles and abstract, n (%)	Screening time (h)^e^
Manual^f^	1.00	1.00	1.00	1.00	1.00	4690 (100)	19
A	0.16 (0.12‐0.21)	0.53 (0.36‐0.70)	0.99 (0.98‐0.99)	0.21 (0.13‐0.31)	0.98 (0.98‐0.99)	830 (17.7)	4.3
B	0.23 (0.18‐0.28)	0.70 (0.52‐0.83)	0.99 (0.98‐0.99)	0.24 (0.16‐0.34)	0.98 (0.98‐0.99)	798 (17.0)	5.5

a The dataset consisted of 4690 titles and abstracts from a previously published scoping review on how AI can support quality of life in people with dementia, covering studies published between 2010 and January 2024.

b The CIs were calculated using Wilson score intervals.

c Level 1 sensitivity was calculated using the 283 titles and abstracts selected for full-text review during manual screening as the reference standard.

d Level 2 sensitivity, specificity, precision, and accuracy were calculated using the 30 titles and abstracts included in the final review as the reference standard.

e Screening time refers only to title and abstract screening and does not include full-text screening.

f Manual screening decisions from the original scoping review served as the reference standard; therefore, values of 1.00 for the manual approach are fixed by definition and should not be interpreted as evidence of perfect manual screening performance.

At level 1, agreement between manual screening and ASReview was moderate for both ASReview approaches (κ=0.24, *P*_o_=0.94 for approach A; κ=0.24, *P*_o_=0.95 for approach B). At level 2, Cohen κ values increased slightly (κ=0.28 for approach A; κ=0.35 for approach B) with high observed agreement (*P*_o_=0.98 for both). McNemar tests showed a significant but level-dependent directional imbalance (*P*<.001). At level 1, ASReview missed more manually selected titles and abstracts than it additionally labeled as relevant: 237 vs 32 for approach A and 219 vs 23 for approach B. At level 2, the direction was reversed: ASReview labeled more titles and abstracts not included in the final review as relevant than it missed final included titles and abstracts, with 62 vs 14 for approach A and 66 vs 9 for approach B. Agreement statistics are presented in Table 2, with the corresponding 2 × 2 contingency tables provided in Multimedia Appendix 1. Analysis of the false negatives revealed no dominant thematic pattern; false-negative abstracts covered both AI and dementia, with no specific topic being consistently missed.

**Table 2.** Agreement and directional imbalance between two ASReview approaches and manual title and abstract screening from a dementia care scoping review.^a^

Level^b^	Comparison^c^	Cohen κ^d^	Observed agreement (*P*_o_)^d^	b^e^	c^e^	McNemar test (*P* value)^f^
1	Manual vs A	0.24	0.94	237	32	<.001
1	Manual vs B	0.24	0.95	219	23	<.001
2	Manual vs A	0.28	0.98	14	62	<.001
2	Manual vs B	0.35	0.98	9	66	<.001

a The dataset consisted of 4690 titles and abstracts from a previously published scoping review on AI-based approaches to support quality of life in people with dementia, covering studies published between 2010 and January 2024.

b At level 1, titles and abstracts selected for full-text review during manual screening were considered relevant. At level 2, titles and abstracts included in the final scoping review were considered relevant.

c Manual screening decisions from the original review served as the reference standard.

d Agreement between each ASReview approach and manual screening was assessed using Cohen κ and observed agreement (*P*_o_).

e The values b and c represent discordant classifications between manual screening and ASReview.

f The McNemar test was used to assess directional imbalance in discordant classifications.

## Discussion

### Principal Findings

This study evaluated the performance of two AI-assisted screening approaches within ASReview compared with manual title and abstract screening in a previously completed dementia care scoping review. Both ASReview approaches substantially reduced screening time and the number of titles and abstracts that required screening, but neither approach retrieved all studies included in the final scoping review. Approach B (custom configuration), which used a semantically richer feature extraction method and a larger prior knowledge set, performed better than approach A (default configuration). However, approach B still missed several final included studies. These findings illustrate that ASReview can support workload reduction in the title and abstract screening process. However, the use of ASReview may be insufficient for reviews in which the aim is to identify and capture as much evidence as possible.

The finding that ASReview can reduce screening workload is in line with previous evaluations of active learning-based screening tools for title and abstract screening [13,14,17,22,35,36]. However, there is no solid overview of how well active learning-based screening tools perform in the title and abstract screening process [23], and previous evaluations show that performance varies across review topics, dataset size, model configurations, prior knowledge strategies, and stopping rules [7,19,37]. This means that ASReview performance cannot be inferred from previous evaluations alone but should be assessed within the specific review context in which it is used. For active learning-based screening workflows, this has implications: a fixed minimum screening proportion, such as 10% or 50% of the total dataset, cannot by itself ensure full identification of relevant titles and abstracts [19,38]. Sensitivity also depends on the model configuration and the composition of the prior knowledge set [19,36]. All these implications may be particularly important in review topics with heterogeneous terminology, where relevant titles and abstracts may be described indirectly or inconsistently [19].

The high specificity and accuracy observed for both ASReview approaches should be interpreted in light of the highly imbalanced dataset. In title and abstract screening, irrelevant titles and abstracts typically form the large majority of the dataset [39]. As a result, high specificity and accuracy can mainly reflect the correct exclusion of irrelevant titles and abstracts rather than the successful identification of relevant titles and abstracts [40]. Therefore, sensitivity, false negatives, and the number of missed final included studies are particularly important for assessing whether an AI-assisted screening workflow is suitable for evidence synthesis.

The 70% (21/30) sensitivity observed for the best-performing ASReview approach represents a critical limitation for using the evaluated workflow as a screening strategy. Approach B retrieved 21 of the 30 final included studies from the original review, meaning that 9 studies would have been missed if this approach had been used as the primary screening approach during the original review. This level of sensitivity would be insufficient for reviews in which near-complete capturing of evidence is expected [41]. By comparison, dual-reviewer screening of titles and abstracts misses around 2.5% of the relevant titles and abstracts [42]. However, dual-reviewer screening is not always feasible because it requires substantial resources [5,13,15]. This trade-off could be acceptable for rapid reviews, where reviewers are willing to give up some degree of certainty regarding the comprehensiveness of the included evidence [42]. However, it remains unclear what level of reduced sensitivity is acceptable when AI-assisted screening tools are used. Further research is therefore needed to determine what level of reduced sensitivity is acceptable when using AI-assisted screening tools in different review contexts.

Agreement analysis highlighted the effect of class imbalance in title and abstract screening. Observed agreement between ASReview and manual screening was high; this was largely driven by shared exclusions of titles and abstracts. In contrast, Cohen κ values ranged from 0.24 to 0.35, which indicate poor agreement beyond chance [43]. This suggests that, despite high observed agreement, ASReview showed limited agreement with manual screening on which titles and abstracts should be selected as relevant. This is expected in title and abstract screening, where relevant titles and abstracts are typically a small proportion of the dataset, but the low kappa values should not be interpreted as acceptable agreement [39,43,44]. The McNemar test provided complementary information by showing that the direction of discordance differed between evaluation levels [45]. At level 1, ASReview missed more titles and abstracts selected for full-text review by manual screening than it additionally labeled as relevant. At level 2, the direction was reversed: ASReview labeled more titles and abstracts that were not included in the final review as relevant than it missed among the final included titles and abstracts. Together, these findings show that high observed agreement could mask practically important disagreement. Missed final included titles and abstracts may reduce the completeness of the evidence base, whereas level 2 false-positive titles and abstracts indicate that workload reduction was accompanied by limited precision among ASReview-selected titles and abstracts.

Current study findings should be interpreted in the context of a broader movement toward responsible use of AI in evidence synthesis. The RAISE (Responsible Use of AI in Evidence Synthesis) recommendations, developed by Cochrane, among others, provide a framework for ensuring the responsible use of AI across all roles within the evidence synthesis process [46]. Similarly, PRISMA-trAIce frameworks provide a reporting framework to improve transparency when AI is used as a methodological tool in evidence synthesis [27]. These developments may support broader adoption of AI-assisted screening tools in the title and abstract screening process but also increase the need for transparent reporting and validation of how such tools are used in specific review contexts [37]. Retrospective validation studies may help identify context-specific best practices for using active learning during title and abstract screening [47]. However, a lack of dataset availability can hinder reproducibility [23,33]. The present study contributes to this evidence by evaluating ASReview’s performance in the context of a dementia care scoping review and by adopting an open science approach, making the full dataset and reviewer decisions publicly available so that the current study is reproducible.

### Strengths and Limitations

To our knowledge, this is the first study to evaluate the performance of ASReview in the dementia care literature research. By making the full title and abstract dataset and all screening decisions available, this study also contributes to transparency, reproducibility, and future validation of AI-assisted screening in this review context. Another strength is that the same researcher conducted both the manual and AI-assisted screening, which helped ensure consistent adherence to the eligibility criteria across all screening approaches. However, the current results need to be viewed in light of some possible limitations. This study focused on a single scoping review, which limits generalizability to other review topics. Second, the manual screening and final inclusion decisions from the original scoping review were used as the reference standard for evaluating both ASReview approaches. Although the original review included an initial dual-screening validation procedure, the final screening decisions should not be interpreted as an error-free gold standard. Third, the ASReview screening approaches were conducted retrospectively by a reviewer who had been involved in the original scoping review title and abstract screening process. Therefore, the reviewer may have been more familiar with the review topic, eligibility criteria, and terminology during the ASReview screening. This familiarity may have influenced labeling decisions and may also have reduced the time needed to assess titles and abstracts compared with the original manual screening process, in which the reviewer still had to become familiar with the literature and screening criteria. Finally, this evaluation was conducted using ASReview version 1.3. The exact extension package name and version used to enable sBERT-based feature extraction in approach B were not retained. Because ASReview and its extensions are under active development, findings may differ with newer releases, plugin versions, alternative model configurations, or updated workflow options.

### Conclusions

This study evaluated two AI-assisted screening approaches within ASReview against manual title and abstract screening in a completed dementia care scoping review. Both ASReview approaches reduced screening time and the number of titles and abstracts requiring manual screening, but neither approach retrieved all titles and abstracts included in the final review. These findings suggest that AI-assisted screening tools, in particular ASReview, can support workload reduction in title and abstract screening but that both evaluated ASReview approaches may be insufficient for reviews in which near-complete capturing of evidence is required. Further research is needed to determine how AI-assisted screening tools perform in review contexts and to determine what level of reduced sensitivity is acceptable when using AI-assisted screening tools in different review contexts.

## Supplementary material

10.2196/84989 Multimedia Appendix 1 Source datasets and screening decision files for the comparative evaluation of 2 AI-assisted screening approaches within ASReview compared with manual screening.

## Abbreviations

ACM: Association for Computing Machinery
PRISMA-trAIce: Preferred Reporting Items for Systematic Reviews and Meta-Analyses–Transparent Reporting of AI-Assisted Evidence Synthesis
RAISE: Responsible Use of AI in Evidence Synthesis
SAFE: Screen, Apply, Find, Evaluate
SBERT: Sentence-Bidirectional Encoder Representations from Transformers
TF-IDF: Term Frequency–Inverse Document Frequency
