# Supplementary material for: Performance of Two AI Approaches in ASReview Compared With Manual Screening for Dementia Care Literature Screening: Comparative Analysis
Source: JMIR Form Res. 2026 Aug 20;10:e84989. doi: 10.2196/84989 (PMC13492632; doi:10.2196/84989)
Supplement: Supplementary file 1 [file formative-v10-e84989-s001.zip › Multimedia/Multimedia_Appendix_README.docx]

**README – Multimedia Appendix**

**Purpose of this appendix**

This Multimedia Appendix provides the datasets and screening decision files used for the comparative evaluation of two AI-assisted screening approaches within ASReview against the manual screening approach from the original scoping review.

The appendix is intended to support transparency and reproducibility by making available the original title-and-abstract dataset, the manual screening reference sets, the ASReview screening decisions, and the prior knowledge records used for both ASReview approaches.

**Overview of included files**

This appendix contains five source files:

1. **Title_and_abstract_screening_manual_approach.enl**
   EndNote library containing the original title-and-abstract dataset used in the manual screening approach. This dataset contains the 4,690 titles and abstracts after deduplication and preparation.
2. **full_text_screening_manual_approach.enl**
   EndNote library containing the references selected for full-text screening in the manual approach.
3. **final_included_reference_manual_approach.enl**
   EndNote library containing the references that were finally included in the original scoping review after full-text screening.
4. **ASReview_approach_A_decisions.xlsx**
   Excel file containing the screening output for ASReview Approach A. The first worksheet contains all screening decisions generated during the ASReview project. The second worksheet contains the prior knowledge records used to initialise Approach A.
5. **ASReview_approach_B_decisions.xlsx**
   Excel file containing the screening output for ASReview Approach B. The first worksheet contains all screening decisions generated during the ASReview project. The second worksheet contains the prior knowledge records used to initialise Approach B.

**Description of the manual screening files**

The manual screening files represent the reference data from the original scoping review.

- The title-and-abstract screening file contains all records available for title-and-abstract screening (after deduplication).
- The full-text screening file contains the subset of titles and abstracts selected for full-text review.
- The final included reference file contains the studies included in the final scoping review.

These manual screening files were used as the reference standard for evaluating the performance of the two ASReview approaches.

**Description of the ASReview files**

The two ASReview Excel files contain the screening decisions for the two AI-assisted screening approaches evaluated in the study.

**Approach A** used the default ASReview configuration with minimal prior knowledge input.

**Approach B** used a custom ASReview configuration with a semantically richer model and a larger prior knowledge set.

For each approach, the Excel file contains:

- a worksheet with the screening decisions generated during the ASReview process;
- a worksheet with the titles and abstracts used as prior knowledge before active learning screening started.

**Relationship between the manual and ASReview datasets**

The manual screening files and ASReview files refer to the same underlying title-and-abstract dataset from the original scoping review. The manual screening decisions and final inclusion decisions served as the reference standard. The ASReview decisions were compared with this reference standard to assess sensitivity, specificity, precision, accuracy, agreement, and screening time.

The comparison was performed at two levels:

1. **Level 1: Title-and-abstract screening level**
   Records selected for full-text review in the manual approach were treated as relevant. Records excluded during manual title-and-abstract screening were treated as irrelevant.
2. **Level 2: Final inclusion level**
   Records included in the final scoping review were treated as relevant. All other records were treated as irrelevant.
